# Loeffler Endocarditis and Associated Parasitosis: A Diagnostic Challenge

**DOI:** 10.7759/cureus.8152

**Published:** 2020-05-16

**Authors:** Htun Latt, Barbara Mantilla, Dwe San, Erwin E Argueta-Sosa, Nandini Nair

**Affiliations:** 1 Cardiology, Texas Tech University Health Sciences Center, Lubbock, USA; 2 Internal Medicine, Texas Tech University Health Sciences Center, Lubbock, USA; 3 Pathology, Texas Tech University Health Sciences Center, Lubbock, USA

**Keywords:** loeffler endocarditis, toxocara canis, restrictive cardiomyopathy, hypereosinophilic syndrome

## Abstract

Loeffler endocarditis is relatively under-recognized and can impose a diagnostic challenge. We present a case of Loeffler endocarditis where eosinophilia was associated with parasitosis. This case highlights the importance of clinical clues in a patient with restrictive cardiomyopathy, and appropriate ancillary testing which helps guide further management.

## Introduction

Loeffler endocarditis is a form of restrictive cardiomyopathy and is possibly an advanced stage of eosinophilic myocarditis (EM). EM constitutes 6% of all types of myocarditis and is a potentially fatal form of myocardial inflammation associated with peripheral eosinophilia [1]. It is a rare diagnosis with most cases being discovered post-mortem [1].

## Case presentation

**Figure 1:**
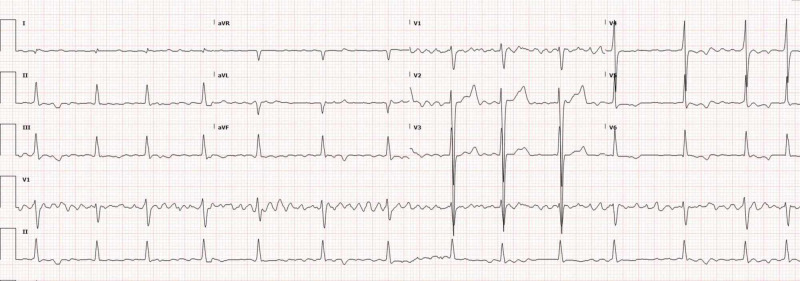
Electrocardiogram showing atrial fibrillation

A 24-year-old Hispanic man presented with intermittent exertional dyspnea, palpitations, and pleuritic chest pain. Symptoms developed approximately within three months, limiting his daily activities. He had moved to the United States from Central America two months prior. Physical examination was significant for an irregularly irregular rhythm. He denied any prior medical history. Two first-degree relatives suffered from heart-related diseases, one of them had a sudden cardiac death. The electrocardiogram (EKG) showed rate-controlled atrial fibrillation (Figure 1).

**Figure 2:**
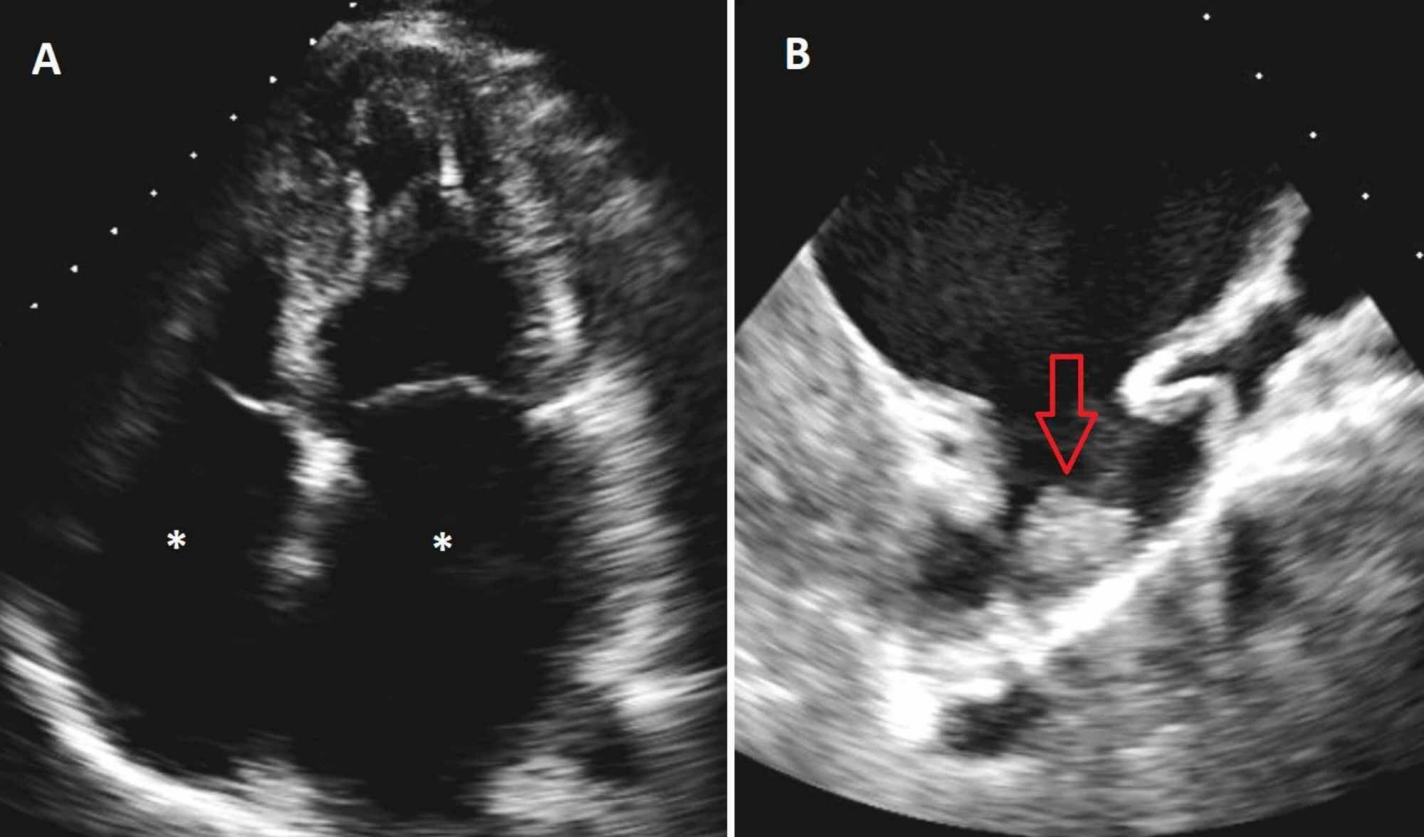
Transthoracic echocardiogram showing bi-atrial enlargement (white asterisks) (A), and transesophageal echocardiogram showing a left atrial appendage thrombus (red arrow) (B)

**Figure 3:**
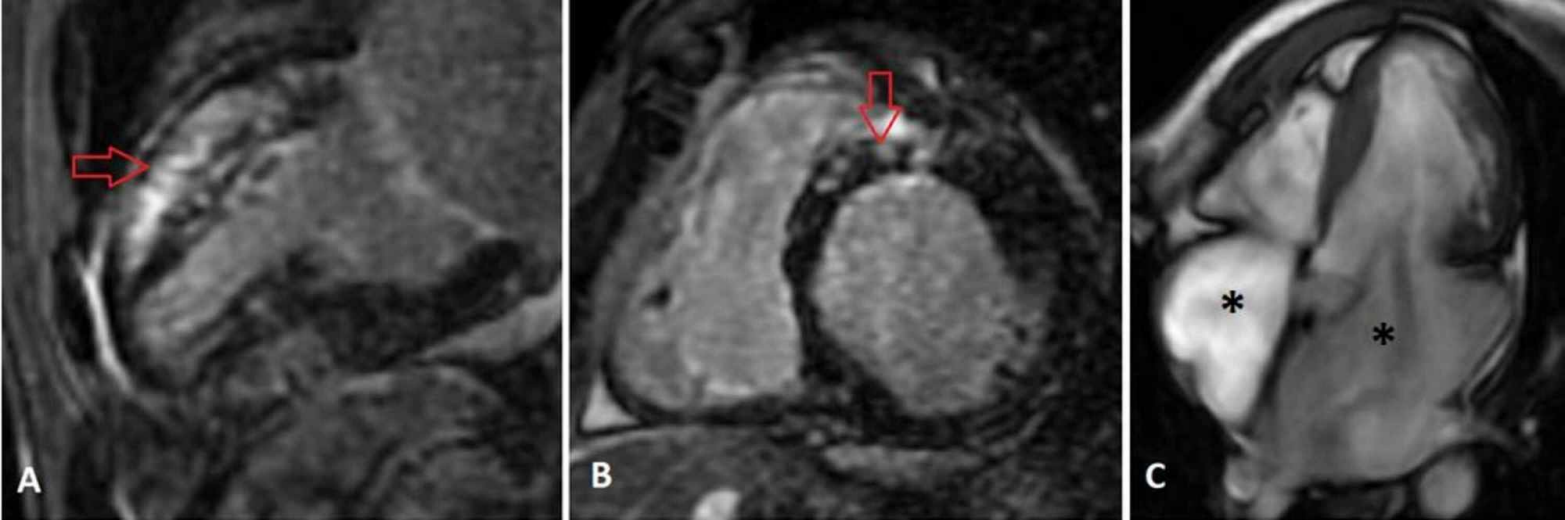
Cardiac magnetic resonance imaging showing abnormal delayed enhancement at subepicardial and mid-myocardial anterior and anteroseptal walls (red arrows), two chamber (A) and short axis views (B). Steady state free precession (SSFP) showing bi-atrial enlargement (black asterisks) (C)

Hematologic testing was significant for peripheral eosinophilia with an absolute eosinophilic count (AEC) of 1.8 K/µL, alanine aminotransferase 101 IU/L, aspartate aminotransferase 113 IU/L, hyperbilirubinemia 1.6 mg/dl, an elevated pro-B-type natriuretic peptide (pro-BNP) of 7704 ng/L, and undetectable troponin T level. Transthoracic echocardiography revealed a normal left ventricular systolic function with a bi-atrial enlargement (Figure 2A). A transesophageal echocardiogram revealed a clot in the left atrial appendage (Figure 2B). Cardiac magnetic resonance imaging (CMR) revealed delayed gadolinium enhancement at the subepicardial and mid-myocardial anterior and anteroseptal walls (Figure 3).

**Figure 4:**
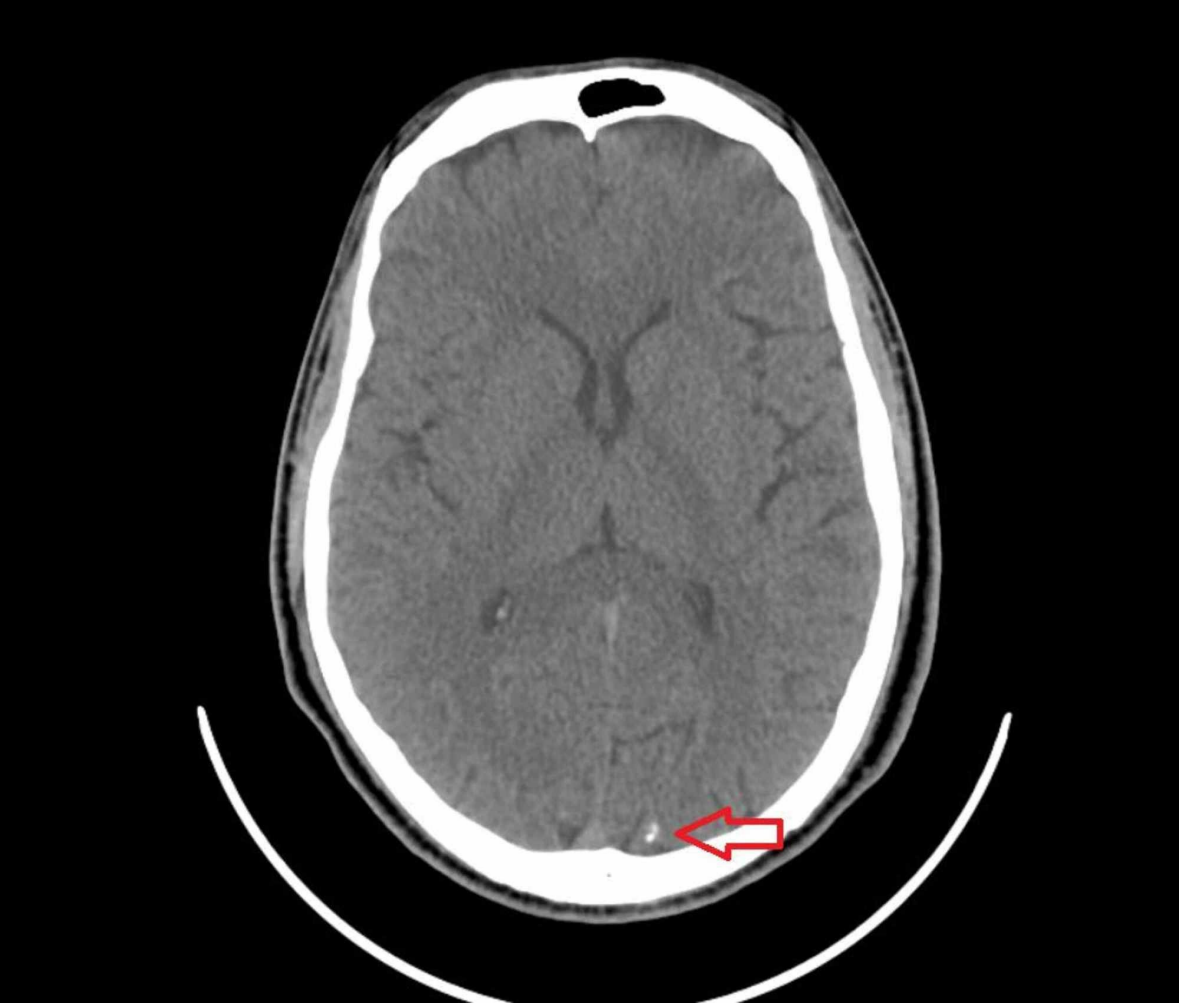
Computed tomography of the head showing a small focus of intraparenchymal calcification in the left occipital lobe

Further testing revealed positive antibodies for Toxocara. Serologic studies for malaria, strongyloidiasis, Chagas disease, and toxoplasmosis were negative. Stool studies were negative for parasitic ova or helminths. Immunoglobulin E level was 1293 IU/ml. Human immunodeficiency virus screen was non-reactive. Serum and urine protein electrophoresis and serum-free light chains were noncontributory. Computed tomography of the head revealed a small focus of intraparenchymal calcification in the left occipital lobe, possibly suggestive of old neurocysticercosis (Figure 4).

**Figure 5:**
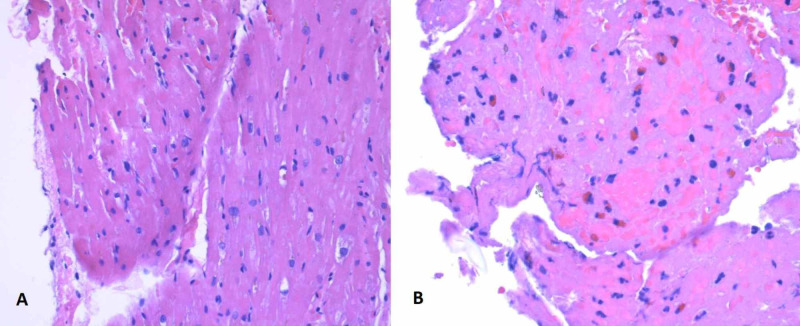
Histopathologic pictures showing myocyte hypertrophy with mild interstitial fibrosis (A) and thrombus with eosinophils (B) in medium magnification (200x)

A right heart catheterization revealed the following pressures (mmHg): right atrial 6, right ventricular (RV) 38/0, pulmonary artery 34/17 (mean 25) and pulmonary capillary wedge 18, and a cardiac index of 2.4 L/min/m2. RV endomyocardial biopsy (EMB) revealed fibrin rich clot in the endocardial surface containing eosinophils (Figure 5). Incidentally, pathogenic heterozygous MYH7 variant was reported in his genetic panel. This variant gene is associated with neuromuscular conditions, which the patient denied. A bone marrow aspirate showed myeloid hyperplasia, one lymphohistiocytic aggregate, and eosinophilia; there was no evidence of monoclonality, suggesting against primary hypereosinophilic syndrome. Testing for bcr-abl and FIP1L1-PDGFRα fusion genes were negative. No C-KIT, Janus kinase 2 (JAK-2) mutations were identified. 

**Figure 6:**
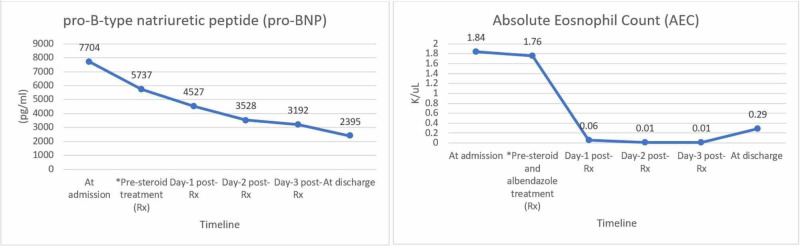
Pro-BNP and AEC pre- and post- steroid/albendazole treatment

Based on the clinical picture, with peripheral eosinophilia, positive Toxocara serology, biatrial enlargement in echocardiography, and RV histopathologic report, our patient was diagnosed with Loeffler endocarditis, associated with parasitic infection. While EMB and ancillary testing results were pending, the patient was started on empiric treatment for myo-pericarditis with indomethacin and colchicine. Due to atrial fibrillation, metoprolol and warfarin had been started. After diagnosis, IV methylprednisolone and albendazole were initiated. Methylprednisolone was later changed to prednisone. After steroid and anti-helminthic therapy, symptoms improved drastically within 48 hours with a drop in AEC and pro-BNP (Figure 6). The patient was discharged with albendazole for a total of four weeks with a gradual taper of prednisone during this time period. He was lost to follow up after discharge.

## Discussion

Eosinophilic myocarditis (EM) constitutes 6% of all types of myocarditis [2]. It is a rare, yet a potentially fatal form of myocardial inflammation associated to peripheral eosinophilia [1]. Pathogenesis involves three different stages: acute myocardial necrosis, thrombosis, and endomyocardial fibrosis which leads to restrictive cardiomyopathy [3,4]. During the acute phase, patients often remain clinically silent. However, eosinophilic necrotizing myocarditis can have a fulminant course [1,5]. In the second or thrombotic phase, eosinophilic granule contents play a major role in thrombus formation along the damaged endocardium giving rise to possible embolic complications [4]. Thirdly, fibro-inflammatory remodeling leads to myocardial and valvular fibrosis with subsequent atrioventricular valve dysfunction and restrictive heart disease [6]. Our patient presented with atrial arrhythmias in the setting of bi-atrial enlargement suggestive of restrictive cardiomyopathy, which corresponds to the later stages of the disease or Loeffler endocarditis.

A systematic revision on all published histologically proven EM cases until June 2017 (n=179) showed that an associated systemic disorder was identified in 64.3% of cases, with hypersensitivity reactions being the most frequently associated condition, followed by eosinophilic granulomatosis with polyangiitis and hypereosinophilic syndrome. Other less frequent causes were infections (n= 9, 5%) mainly attributed to *Toxocara canis* (*T. canis*) (n= 6, 3.4%) [1]. Hypereosinophilic syndrome can be further categorized into primary (neoplastic or clonal), secondary (reactive as seen in parasitosis), and idiopathic types. Determining the etiology is critical for management. 

Patients with EM can present with a wide spectrum of clinical manifestations from subtle nonspecific symptoms to a life-threatening cardiac arrhythmia. The most common symptoms are dyspnea and chest pain [1,7]. EM predominantly affects males with a median age of 41 years [1,3]. Endocavitary thrombi is associated with persistent eosinophilia and has been reported in 12.3% of histologically proven EM, suggesting a role for anticoagulation during the acute phase [1]. However, absence of eosinophilia at admission does not exclude the diagnosis of EM [1].

Toxocara causes parasitic infection in dogs (*T. canis*) and cats (*Toxocara cati*), that may transmit the roundworm to humans through the fecal-oral route. While most seropositive patients are asymptomatic, some will develop a form of human toxocariasis called visceral larva migrans (VLM), of which 10%-15% may have myocardial involvement [3,8]. Myocarditis can occur from direct larval invasion to the myocardium and/or hypersensitivity reactions [3]. Early treatment with albendazole is recommended in EM associated with VLM [3]. Systemic corticosteroids are also used for treatment due to their effect in inhibiting eosinophilic invasion into the myocardium. Because seropositivity for Toxocara cannot distinguish between active or remote infection and no definitive testing for acute infection is available, empiric therapy with albendazole and prednisone were initiated in our patient [8].

EMB is the gold standard for diagnosis of EM despite its limited sensitivity in focal infiltrative disease [1,3]. CMR is also a helpful non-invasive tool which can identify myocardial inflammation or scar. The most common late gadolinium enhancement pattern in CMR is subendocardial [1].

No clinical trials have tested the efficacy of steroids in patients with EM. Studies have shown that there is a lower incidence of in-hospital death in patients treated with steroids (n=10, 9.9%) compared with those who do not receive steroids (n=23, 65.7%; p=0.0001) [3]. Steroids have been shown to decrease acute inflammation and prevent progression to necrosis. CMR studies of patients with EM post-steroid treatment demonstrate a reduction in subendocardial gadolinium enhancement [3]. Patients with chronic EM also benefit from glucocorticoid treatment as evidenced in our case. Surgical intervention may be helpful especially in late fibrotic disease involving valvular structures [7]. EM has been associated with in-hospital mortality rate of 22.3% [1]. No data is available on recurrence. Though in controversy, immunosuppressive therapy may prevent recurrence [5].

## Conclusions

The diagnosis of Loeffler endocarditis can be challenging. In the appropriate clinical setting, low threshold of clinical suspicion should be maintained. EMB is considered the gold standard for diagnosis and should be performed with adequate clinical suspicion. Etiology of eosinophilia should be determined as treatment can be targeted to a specific cause.
